# Using Machine Learning for Non-Invasive Detection of Kidney Stones Based on Laboratory Test Results: A Case Study from a Saudi Arabian Hospital

**DOI:** 10.3390/diagnostics14131343

**Published:** 2024-06-25

**Authors:** Hanan Alghamdi, Ghada Amoudi

**Affiliations:** Department of Information Systems, Faculty of Computing and Information Technology, King Abdulaziz University, Jeddah 21589, Saudi Arabia; gaamoudi@kau.edu.sa

**Keywords:** kidney stone, machine learning in healthcare, medical diagnostics, predictive modeling, imputation, laboratory tests, imbalanced dataset, XGboost, random forest

## Abstract

Kidney stone disease is a widespread urological disorder affecting millions globally. Timely diagnosis is crucial to avoid severe complications. Traditionally, renal stones are detected using computed tomography (CT), which, despite its effectiveness, is costly, resource-intensive, exposes patients to unnecessary radiation, and often results in delays due to radiology report wait times. This study presents a novel approach leveraging machine learning to detect renal stones early using routine laboratory test results. We utilized an extensive dataset comprising 2156 patient records from a Saudi Arabian hospital, featuring 15 attributes with challenges such as missing data and class imbalance. We evaluated various machine learning algorithms and imputation methods, including single and multiple imputations, as well as oversampling and undersampling techniques. Our results demonstrate that ensemble tree-based classifiers, specifically random forest (RF) and extra tree classifiers (ETree), outperform others with remarkable accuracy rates of 99%, recall rates of 98%, and F1 scores of 99% for RF, and 92% for ETree. This study underscores the potential of non-invasive, cost-effective laboratory tests for renal stone detection, promoting prompt and improved medical support.

## 1. Introduction 

Kidney stone disease is one of the most widespread urinary tract conditions, especially in hot climates such as Saudi Arabia, with a high prevalence of 20% of the population. Kidney stones often occur in males more than in females (ratio: 3.2:1) [1]. Timely and accurate diagnosis of kidney stones is crucial to prevent severe complications such as chronic kidney disease and renal failure. However, traditional diagnostic methods, primarily computed tomography (CT) scans, pose significant challenges due to their high cost, radiation exposure, and delays in obtaining radiology reports.

Machine learning (ML) is rapidly becoming integral to the modern healthcare industry, providing tremendous potential for diagnosis, prognosis, and epidemiology, as opposed to traditional methods, which rely on self-learning and lengthy experience [2]. ML involves using algorithms and statistical models to enable computer systems to learn from data without being explicitly programmed. One of the main advantages of ML in disease diagnosis is the ability to identify diseases early. Early detection of chronic conditions can lead to more effective treatments. ML can also aid in disease management by providing real-time updates on disease progression. Moreover, with the increasing population and shortage of healthcare personnel, ML is highly cost-effective. Therefore, it is becoming increasingly vital to develop diagnostic tools that can automate medical procedures to make accurate diagnoses and speed up the diagnostic process. 

Laboratory tests are one of the primary diagnostic tools used in healthcare to help detect the presence of a disease or determine the severity of an illness. These tests may involve the collection of blood, urine, or other bodily fluids to determine the presence of pathogens, infections, allergies, or other health conditions. Each type of test can provide unique information about the patient’s health status and can help to guide the treatment process. However, the interpretation of laboratory test results can be challenging due to the complexity and variability of the data. Machine learning algorithms can help overcome these challenges by identifying unique data features and making accurate predictions. 

Despite their potential, the application of ML in medical diagnostics is often hindered by incomplete and imbalanced clinical datasets. Missing values in clinical data can lead to biased estimates and reduced accuracy, making it imperative to employ effective techniques to handle missing data. Several approaches can be used, including deletion, which excludes incomplete records, and data imputation. Data imputation involves filling in missing values with estimated or predicted data while minimizing the impact on other variables in the dataset. 

Several approaches for imputing missing data exist, varying from simple to advanced and complicated procedures [3]. Simple methods for imputing missing data include mean imputation, regression imputation, and k-nearest neighbor imputation. Mean imputation is the simplest of the techniques and involves replacing missing values with the mean value of the corresponding feature. K-nearest neighbor imputation estimates missing values based on the values of the k-nearest neighbors of the missing value in the feature space [4]. Multiple imputations (MI) is a more sophisticated approach involving replacing missing values with estimated ones [5]. For example, the random forest (RF) imputation approach uses a forest of decision trees to estimate missing values in a dataset. The method works by creating multiple decision trees on the observed data and then using these trees to predict the missing values. The imputed values are based on the averages of the predictions from all the decision trees in the forest. This process is repeated multiple times to generate a final imputed dataset. Multiple imputations by chained equations (MICE) is another widely used approach [6,7]. MICE uses a series of regression models to impute missing values. It is based on the principle of creating multiple imputations for missing values by random draws from a predictive distribution. MICE creates several imputations for each missing value and uses statistical tools to combine these imputations to produce a final estimate. MICE allows the imputation of different variable types that may reside in the data by regressing each variable’s predicted distribution on all other variables to approximate it. 

This study aims to explore the potential of ML in detecting renal stones using laboratory test results, addressing the challenges of missing data and imbalanced class distribution. We evaluate various ML algorithms and imputation techniques to identify the most effective approach for early renal stone detection. Our research leverages a comprehensive dataset from a Saudi Arabian hospital, the largest of its kind, to demonstrate that laboratory tests can serve as a reliable, non-invasive, and cost-effective means of identifying renal stones. This innovative approach promises to enhance urological diagnostics, ensuring timely and improved medical support for patients. 

The rest of this paper is organized as follows—Section 2 reports related works using laboratory test results to diagnose kidney diseases and handle missing values. In Section 3, the proposed methodology has been detailed. In Section 4, the results obtained are analyzed. In Section 5, concluding remarks have been presented. 

## 2. Related Work

Some studies have explored the application of laboratory tests to predict kidney-related diseases—for example, studies [8,9] utilized ML to predict kidney failure. Authors in [9] proposed a framework to predict renal failure using five ML algorithms. XGBoost achieved the highest performance with an AUC of 91%. In [8], the authors attempted to predict when patients must be dialyzed and receive personalized care. A set of extremely randomized trees classifiers considering 27 features was proposed, and an accuracy of 94% was obtained in predicting the development of renal failure. 

Authors in [10] used clinical, demographic, and laboratory data of patients admitted to acute care units in a large tertiary care hospital to identify patients with kidney stones. Patients diagnosed with kidney stones were compared to groups with acute abdominal pain and other conditions. The results show that, among 8597 patients, 217 were identified with kidney stones and 7556 with acute pain due to other causes. A sensitivity of 81% and a specificity of 86% were obtained. This study analyzed a sample from a single site without external validation, and a high-class imbalance was observed in the used data sample [10]. Kazemi and Mirroshandel [11] proposed an ensemble learner model to predict kidney stone type, applying the learner to data collected from kidney patients between 2012 and 2016. The dataset had 42 features processed on WEKA using different classifiers, such as Bayesian models, decision trees, rule-based classifiers, and artificial neural networks (ANNs). The ensemble-based model achieved 97% accuracy. Halder et al. [12] developed a web-based kidney disease prediction application using a publicly available dataset from the UCI machine learning repository. The features used include demographic, clinical, and laboratory data. The study applied various machine learning models, with AdaBoost being the best-performing model. Using laboratory data and a set of novel predictive features, such as the use of diabetic medication, Nguycharoen [13] created an explainable machine-learning model to predict chronic kidney diseases in cardiovascular patients. The study applied the random forest model, claiming it has a high sensitivity rate, 0.882, which reduces false negatives, as the primary use of this model was for screening purposes. 

Other studies [14,15,16] tested the KNN imputation method, which was implemented for data preprocessing to impute the missing values. Some researchers utilized MICE [17], where the authors used a large dementia diagnosis dataset. They tested various imputation techniques, including SI and MI. When tested against the complete dataset, MICE and RF imputation provided the highest accuracy for predicting the mean of the artificially missing datasets. Gupta et al. [18] applied a novel technique to the Alzheimer’s Disease Neuroimaging Initiative data. According to simulations, the coverage probability of a 95% interval derived using MICE can be less than 1. Study [10] developed an ensemble learning model combining six base models. The study used eXtreme gradient boosting as their ensemble to perform imputation on data. They found that their ensemble model outperformed the reference model by 9.69%. In other medical fields, Hegde et al. compared imputation techniques on a dental electronic health record dataset and found that the probabilistic principal component analysis technique yielded better imputation results than the other techniques [19].

It can be concluded that missing data and imbalanced class distribution challenges are unavoidable in clinical research. The exclusion of missing data records may lead to a significant loss of the original dataset, which in turn causes a substantial loss of the ML algorithm’s prediction power. Thus, imputations are commonly used to deal with missing data. However, the choice of the imputation method depends on the nature of the data and the intensity of the missing data. Moreover, classifiers might achieve different results with different imputation and data-balancing techniques. Thus, in this paper, we aim to explore the potential of ML prediction models to accurately predict the stone presence using lab test results while having an imbalanced and sparse dataset. 

## 3. Methodology

**Figure 1:**
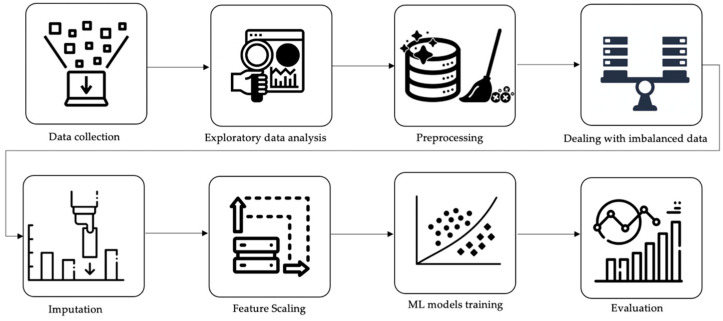
Workflow of the proposed methodology.

This study collected laboratory test results, including radiology reports, for patients with and without kidney stones from the Urology Department at the International Medical Center (IMC) in Jeddah, Saudi Arabia, between 2016 and 2018. The dataset, named the Saudi Renal Stone Prediction (SRSP) dataset, comprised 2156 patient records and 11 lab tests. The steps depicted in Figure 1 were taken to preprocess the data and develop the machine-learning models. The proposed workflow consists of data collection, exploratory data analysis, preprocessing, dealing with imbalanced datasets, experimenting with different imputation techniques, data scaling, ML algorithms training, and evaluating and comparing several approaches. The statistical analysis was performed using Python programming language, version 3.10.12. Specifically, we used libraries such as NumPy, version 1.25.2, for data manipulation, Pandas, version 2.0.3, for data processing, SciPy, version 1.13.1, for conducting statistical tests, and Matplotlib, version 3.7.1, for generating figures and visualizations.. In the following subsections, we briefly discuss all stages of our study. 

### 3.1. Dataset Collection 

The data included various features such as urine pH, serum creatinine, calcium, and magnesium levels. Expert radiologists generated radiology reports to identify the presence of stones in CT scan photographs. The data were de-identified, and new IDs were randomly generated to maintain patient confidentiality.

### 3.2. Exploratory Data Analysis 

At this stage, we explored and visualized the data to identify potential discrimination patterns in the patient’s lab test results and to guide the selection of appropriate ML algorithms. Table 1 describes all features used in this work, including the target label, 11 lab tests, and the patient’s age, gender, and BMI. Several features have substantial missing values, particularly magnesium (Mg) serum, phosphorous serum, and calcium serum, which require imputation techniques to handle missing data. Features like alkaline phosphatase, creatinine serum, red blood cells in urine, and white blood cells in urine have high standard deviations, indicating significant variability among patients. 

**Figure 2:**
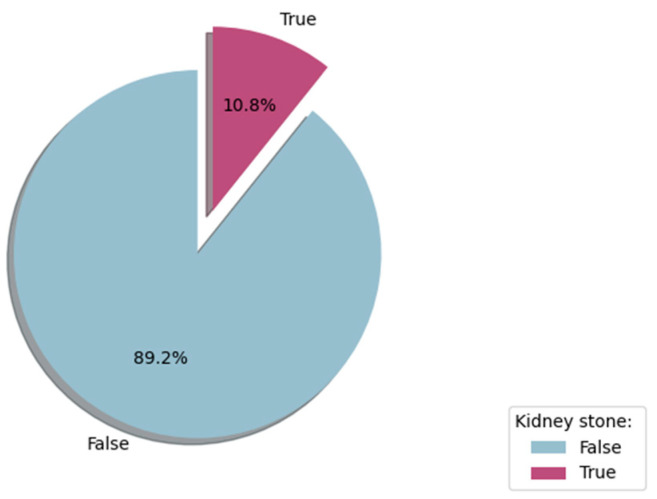
Target label (stone) distribution in the SRSP dataset.

Figure 2 shows the stone presence distribution in the SRSP dataset. This figure highlights the class imbalance in the dataset, with a significantly higher proportion of patients without kidney stones (89.2%) compared to those with kidney stones (10.8%). This imbalance necessitates the use of techniques such as oversampling or undersampling during the data preprocessing phase to ensure balanced training of machine learning models.

**Figure 3:**
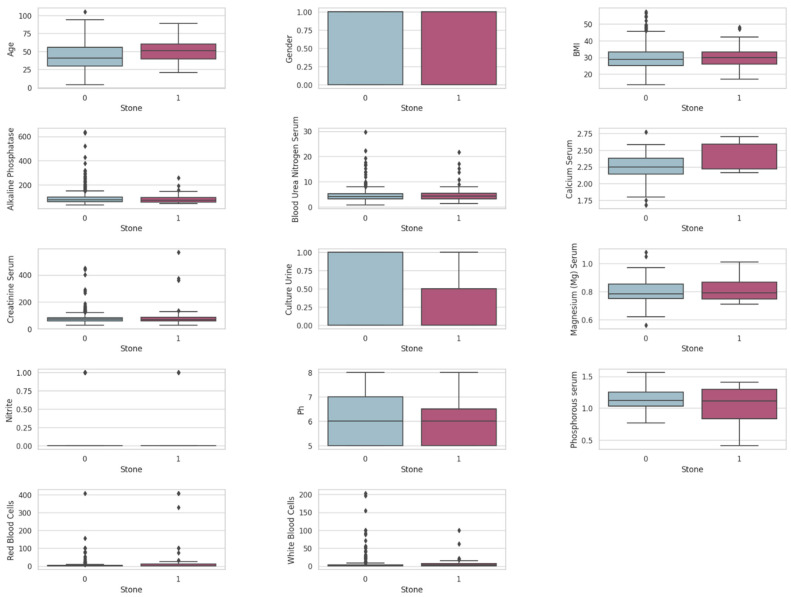
Box plots of the SRSP dataset’s features (0—no stone, 1—stone).

Figure 3 presents the plots that demonstrate the locality, spread, and skewness of the SRSP dataset’s features. These plots use a typical box plot layout where the box represents the interquartile range (the middle 50% of data), the line inside the box marks the median, and the whiskers extend to show the full range of data excluding outliers, which are marked as individual points. Outliers identified in the plot highlight potential anomalies or variations that require careful consideration during data preprocessing. To ensure robust model performance, if the outliers represented extreme but valid observations, they were either removed or transformed to reduce their impact on the model. For example, as shown, some of the creatinine serum levels do not align with the general population. Instead of removing these outliers, which might represent true clinical scenarios, they could be capped at the 95th percentile. This approach reduces the influence of extreme values without completely discarding them. On the other hand, for magnesium (serum) measurements, the detected outliers were likely attributable to measurement errors or exceptionally rare conditions. To address this, we suggested adjusting both the upper and lower extremes to align with the nearest acceptable quantile thresholds to preserve most of the data’s integrity while mitigating the influence of extreme outliers.

**Figure 4:**
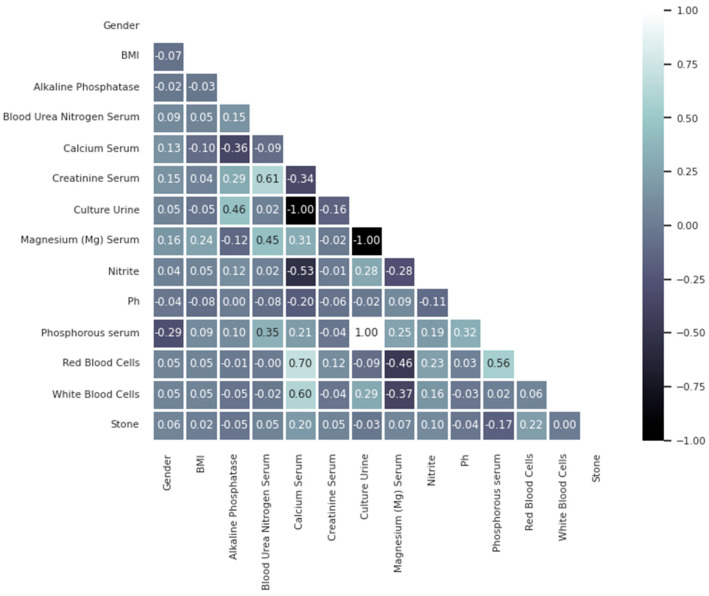
Heatmap correlation of the SRSP dataset’s features.

Figure 4 presents a correlation matrix heatmap, which illustrates the relationships between various features in the SRSP dataset, including the target feature ‘stone’, which indicates the presence of kidney stones. The correlation coefficients range from −1 to 1, indicating the strength and direction of the linear relationship between feature pairs. Most features exhibit weak correlations with each other, suggesting that they are relatively independent. This independence is beneficial as it reduces the risk of multicollinearity, which can adversely affect the performance of machine learning models. However, the weak correlations between the ‘stone’ feature and other features emphasize the need for sophisticated machine learning models that can capture complex, non-linear relationships to accurately predict the presence of kidney stones using laboratory test results. 

### 3.3. Data Preprocessing

Since actual hospital patients’ medical records are unlabeled and uncleaned, preprocessing was essential for creating a well-structured, annotated dataset. There were many redundant and missing values, and some outliers were detected, as shown in Figure 3. Therefore, we performed various preprocessing techniques to clean and organize the data.

First, repeated values were removed to eliminate redundancy. Second, we detected and removed outliers using the interquartile range (IQR) method. Outliers were defined as values falling below Q1 − 1.5IQR or above Q3 + 1.5IQR, and these were carefully reviewed and either corrected or removed if deemed extreme but valid.

Third, since each patient had multiple rows corresponding to the number of laboratory tests (e.g., a patient with three tests would have three records), we restructured the data to ensure one record per patient. This involved aggregating the test results into a single comprehensive record for each patient.

Fourth, some values required encoding. Binary values were converted to 0 and 1 (e.g., “0” for females and “1” for males in the gender feature), and string values were removed from numerical fields (e.g., units in BMI were standardized).

Fifth, the data were labeled using CT scan reports. To ensure accurate data labeling, the manual annotation process was enhanced using Cohen’s Kappa method, where one rater assessed two trials on each sample. This method improved labeling accuracy by ensuring consistency and reliability.

Sixth, to enhance the quality of the results, features were selected for their specificity and relevance, ensuring that irrelevant features did not affect the target variables. The parameters for feature selection were determined based on the expertise of a qualified urologist. Kidney stone occurrence was chosen as the prediction target, with other lab tests serving as predictive features.

### 3.4. Sampling Imbalanced Data

As shown in Figure 2, the dataset suffers from a severe skew in the class distribution. Random oversampling method (ROS) and random undersampling method (RUS) are commonly used techniques to tackle this issue. ROS duplicates examples from the minority class in the training dataset. In contrast, RUS deletes samples from the majority class to balance the dataset. It is worth noting that duplication in ROS can lead to overfitting for some classification models. On the other hand, RUS can result in losing invaluable information to train the ML models. Therefore, we evaluated both techniques to generate proper training data for the ML algorithms. 

### 3.5. Imputation

As shown in Table 1 data missingness was observed in most features. Imputation is a technique that replaces the missing data with some substitute value to retain most of the information in a dataset. We used imputation because removing missing value records is not feasible and will considerably reduce the dataset’s size. However, choosing an appropriate imputation algorithm is essential. Thus, this study evaluated four imputation techniques, including mean, median, k-nearest neighbor (KNN), and multivariant imputation by chained equations (MICE). 

Mean and median imputations are simple and impute the missing values with the mean or median of the feature. In contrast, KNN imputation finds ‘k’ instances in the dataset close to an instance with missing values. Then, KNN uses these ‘k’ instances to estimate the value of the missing feature, typically the mean of its ‘k’-neighbors feature values [3].

Thus, KNN is a similarity-based method that uses distance metrics to measure the similarity between instances. We used Euclidean distance as a metric to evaluate the distance in our work. In case of missing coordinates, the distance is calculated by ignoring the missing values and scaling up the weight of the non-missing coordinates:

(1) $$\mathrm{dist}\left(x,y\right)=\sqrt{\mathrm{weight}\times \mathrm{squared}\;\mathrm{distance}\;\mathrm{from}\;\mathrm{present}\;\mathrm{coordinates}\;}$$

where

(2) $$\mathrm{weight}=\frac{\mathrm{total}\;\mathrm{number}\;\mathrm{of}\;\mathrm{coordinates}}{\mathrm{number}\;\mathrm{of}\;\mathrm{present}\;\mathrm{coordinates}}$$

MICE is a multiple imputation technique that imputes missing values through an iterative set of predictive models, typically with 10 to 20 iterations. In each iteration, MICE imputation uses other feature dimensions in the dataset to predict the missing values in the current feature. Iterations run until the convergence of predictive models is reached [20]. 

### 3.6. Feature Scaling 

**Figure 5:**
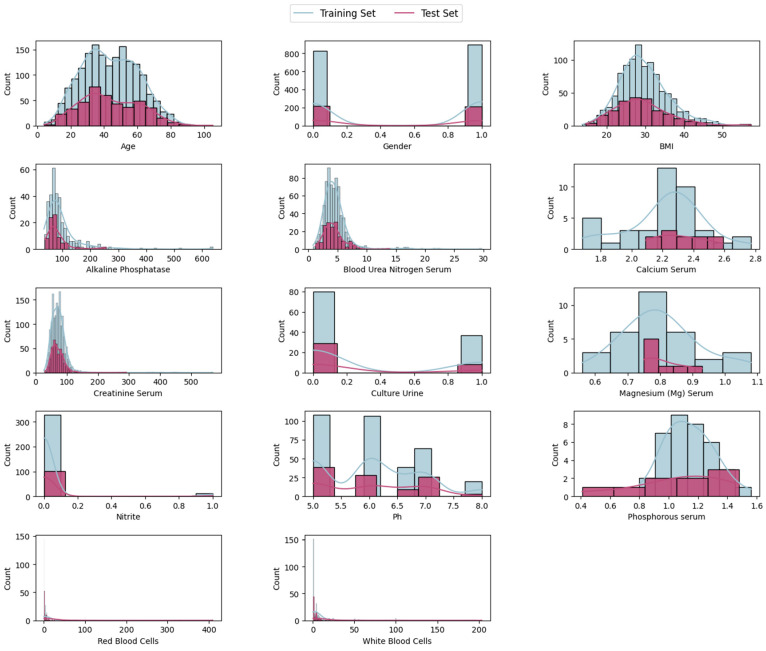
Histogram of the original SRSP dataset’s features for the training and test sets examples.

Features in the SRSP dataset have a wide range of values, as shown in Figure 5. Feature scaling is a required step for many ML algorithms that eases the convergence for other algorithms. In this study, we utilized standardization scaling, also called Z-score normalization, which involves rescaling each feature such that it has a mean of 0 and a standard deviation of 1 using the following formula:

(3) $$\mathrm{NFV}=\frac{\left(x-\mu \right)}{\sigma }$$

where NFV is the new feature value, x is the original value, μ is the mean, and σ is the standard deviation of the feature values. 

### 3.7. ML Classification Models

We explored a variety of classifiers to suggest the most promising predictors. Some classifiers, such as the decision tree (DT), were single-based, while others were ensemble-based, such as the random forest (RF). All classifiers were trained using 40-cross validation splits with 3846 training samples for oversampling and 464 training examples for undersampling. The classifier algorithms applied in this work are as follows: 

#### 3.7.1. Naive Base (NB)

The Naive Bayes classifier is a simple yet powerful algorithm widely used in ML for classification tasks. It is based on the Bayes theorem, which states that the probability of a certain event occurring given a certain set of conditions can be calculated by the product of the conditional probabilities of each attribute given that event:

(4) $$P\left(c|x\right)=\frac{P\left(x|c\right)P\left(c\right)}{P\left(x\right)}$$

where $P\left(c|x\right)$ is the posterior probability of the target class c, given features x, $P\left(c\right)$ is the prior probability of the class c, $P\left(x|c\right)$ is the likelihood, the probability of the feature given the class, and $P\left(c|x\right)$ is the prior probability of the features. Despite its simplistic approach, the NB classifier has been found to perform well in various applications [21]. 

#### 3.7.2. Logistic Regression (LR)

LR is a statistical tool that can model a relationship between multiple features and a binary target variable. LR is used as an input to another function, such as the linear equation:

(5) $$h\theta \left(x\right)=\;g\;\left(\theta^{T}x\right)\;\mathrm{where}\;0\;\leq \;h\theta \;\leq 1$$

where x is the feature column vector, $\theta^{T}$ is the row coefficient vector, and g is the logistic or sigmoid function, which can be given as follows:

(6) $$g\left(z\right)=1/(1+e^{-z})\;\mathrm{where}\;z={\;\theta }^{T}x$$

The resulting model can then predict the probability of the dependent variable taking on a particular value for a given set of features [21]. 

#### 3.7.3. Decision Tree (DT)

A decision tree classifier is a widely used ML algorithm. It is constructed by recursively splitting data into smaller subsets based on feature-based rules, ultimately creating a tree-like structure in which each internal node represents a feature, and each leaf node corresponds to a unique class label. One of the key advantages of a DT classifier is its interpretability and robustness to noisy data. However, a DT is prone to overfitting, resulting in poor generalization performance. This issue can be mitigated by ensembling multiple decision trees. Ensemble methods such as RF and gradient boosting (GB) can improve classification accuracy and reduce the risk of overfitting [21]. 

#### 3.7.4. K-Nearest Neighbors (KNN)

KNN is a non-parametric algorithm that makes predictions based on the instances in the training dataset. KNN looks for the ‘k’ closest instances in the training dataset to the current instance. The predicted class depends upon the classes of the nearest ‘k’ instances in the dataset. Different distance measures can be used with KNN, such as Manhattan distance, Euclidean distance, or Minkowski distance. We used Euclidean distance in this work, as explained previously in [22]. 

#### 3.7.5. Stochastic Gradient Descent (SGD) 

Stochastic gradient descent (SGD) is a popular algorithm for optimizing ML models. SGD aims to minimize the loss function by iteratively updating the model’s parameters. The algorithm works by randomly selecting a subset of the data and computing the gradient on that subset, which updates the model’s parameters. SGD offers several advantages: scalability, fast convergence, and efficiency. However, some challenges are associated with SGD, such as choosing an appropriate learning rate and the possibility of getting stuck in a suboptimal solution [21].

#### 3.7.6. Support Vector Machine (SVM) 

SVMs have become increasingly popular due to their ability to handle high-dimensional and non-linearly separable data. SVM is also robust to outliers and can handle unbalanced data. SVMs use a hyperplane to separate the input data into classes. The maximum margin hyperplane that maximizes the separation of the classes is the maximum margin hyperplane [21]. 

#### 3.7.7. Multi-Layer Perceptron (MLP)

MLP is a powerful neural network model composed of multiple layers of artificial neurons; each layer is connected to the neurons in the adjacent layers. The architecture of an MLP consists of three basic types of layers: the input layer, one or more hidden layers, and the output layer. Each neuron in the input layer represents a single input feature. The hidden layers are responsible for processing the input data and extracting valuable features through a series of nonlinear transformations. The final layer of the network is the output layer, which produces the network’s final output [21].

#### 3.7.8. Random Forest (RF)

RF is an ensemble learning algorithm combining multiple decision trees to produce more accurate and robust predictions. RF builds a set of decision trees using a random subset of features and a random subset of the training data. Each of these trees is trained on a different subset of data, and the final prediction is made by aggregating the predictions of all the trees in the forest [21].

#### 3.7.9. Gradient Boosting (GB)

GB is a type of ensemble learning that combines the predictions made by multiple weak models to form a more accurate final prediction. GB works by building an initial model, then using that model to identify errors and train a new model to correct those errors. This process repeats, with each new model refining the predictions of the previous one until the model is of sufficient accuracy [21].

#### 3.7.10. Extreme Gradient Boosting (XGBoost)

XGBoost is a decision tree-based ensemble learning algorithm that uses a combination of weak learners to create a robust model. It entails building a sequence of decision trees that aim to correct the errors of the previous ones. As a result, the final model is a highly accurate, highly generalized predictive model capable of handling large and complex datasets [14].

#### 3.7.11. Extremely Randomized Trees (ETree) 

An ETree is a type of decision tree-based ensemble learning method. This technique is similar to RF but goes a step further by randomly selecting candidate splits for each tree rather than simply considering all possible splits. The result is a highly diverse set of decision trees that are less correlated with each other, reducing the risk of overfitting and improving overall model performance [14].

It is worth noting that we have chosen ML over deep learning models for this study due to several key factors. Firstly, our dataset size is relatively small, which is more suitable for traditional ML algorithms that perform well with limited data. Deep learning models, on the other hand, typically require large amounts of data to achieve optimal performance. Secondly, ML algorithms are generally less computationally intensive than DL models, making them more accessible and efficient given our available computational resources. Finally, the specific nature of our problem, which involves structured data from laboratory test results, aligns well with the strengths of ML techniques. These algorithms are adept at handling structured data and can provide highly interpretable results, which is crucial for medical diagnostics. 

### 3.8. Evaluation

The dataset was split into 70% for learning and 30% for testing to evaluate all classifier models. We compared the classifiers’ performance using the following metrics: 

#### 3.8.1. Accuracy

The accuracy gives the percentage of correct classifications as follows:

(7) $$\mathrm{Accuracy}=\frac{\;\left(\mathrm{TP}+\mathrm{TN}\right)}{\left(\mathrm{TP}+\mathrm{FP}+\mathrm{FN}+\mathrm{TN}\right)}$$

where TP is the number of truly classified positive class samples, TN is the number of truly classified negative class samples, FP is the number of incorrectly classified negative class samples, and FN is the number of incorrectly classified positive class samples. 

#### 3.8.2. Precision

Precision measures how many samples classified as positive are truly positive.

(8) $$\mathrm{Precision}=\frac{\mathrm{TP}}{\left(\mathrm{TP}+\mathrm{FP}\right)}$$

#### 3.8.3. Recall

Recall measures how many samples are truly classified as positive among all positive samples.

(9) $$\mathrm{Recall}=\frac{\mathrm{TP}}{\left(\mathrm{TP}+\mathrm{FN}\right)}$$

#### 3.8.4. F1 Score

F1 score is a harmonic mean between precision and recall:

(10) $$F1\;\mathrm{Score}=2\times \frac{\left(\mathrm{Precision}\times \mathrm{Recall}\right)}{\left(\mathrm{Precision}+\mathrm{Recall}\right)}$$

#### 3.8.5. ROC and AUC Score 

The receiver operator characteristic (ROC) curve is a probability curve that plots the true positive rate (TPR) against the false positive rate (FPR) at various threshold values. ROC visualizes the performance of a binary classifier. The area under the curve (AUC) is used as a summary of the ROC curve. The higher the AUC, the better the model’s performance. A perfect classifier has an AUC equal to 1. Meanwhile, the random classifier has an AUC equal to 0.5. 

## 4. Experimental Results and Discussion

Since the dataset has a lot of missing values, we applied a variety of imputation methods. First, we conducted simple mean, median, KNN, and MICE imputations. Then, we balanced the data using ROS and RUS. Finally, we evaluated the performance of the eleven classification algorithms described above. 

**Figure 6:**
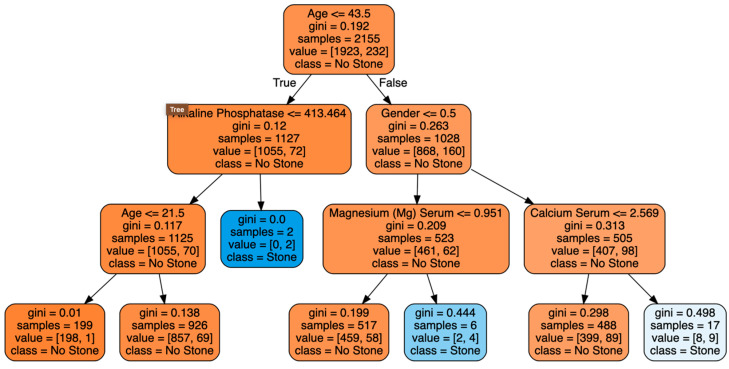
Visualization of the decision tree classifier.

In Figure 6, we visualized the DT to obtain better insight into the importance of the dataset features. The root node highlights age as the most critical factor in predicting kidney stones since it is used for the initial split. Gini impurity assesses the purity of the nodes. A lower Gini score signifies a more uniform node. Nodes with Gini scores near 0 indicate greater confidence in classification, whereas higher Gini scores reflect less certainty. The terminal nodes (leaves) represent the final classifications. For example, a leaf node with a Gini score of 0.0 and samples [0, 2] indicates that all instances in that node are classified as having stones (class = stone).

Other significant features include alkaline phosphatase, gender, magnesium (Mg) serum, and calcium serum. The figure also shows that the DT is not confident at all leaf nodes, as the impurity (Gini) scores are not close to 0. This decision tree reveals that while age is a primary factor, other variables such as alkaline phosphatase, gender, magnesium, and calcium levels also play roles in predicting kidney stones. The differences in Gini scores across the tree suggest that some splits lead to more confident classifications than others, underscoring the dataset’s complexity. This observation motivated us to examine more ensemble classifiers. By using ensemble methods such as random forest, XGBoost, and extremely randomized trees, we aimed to leverage the strengths of multiple decision trees to improve overall prediction accuracy and capture the complex, non-linear relationships within the dataset. Ensemble classifiers are particularly effective in enhancing model robustness and discovering critical features that a single decision tree might overlook.

**Figure 7:**
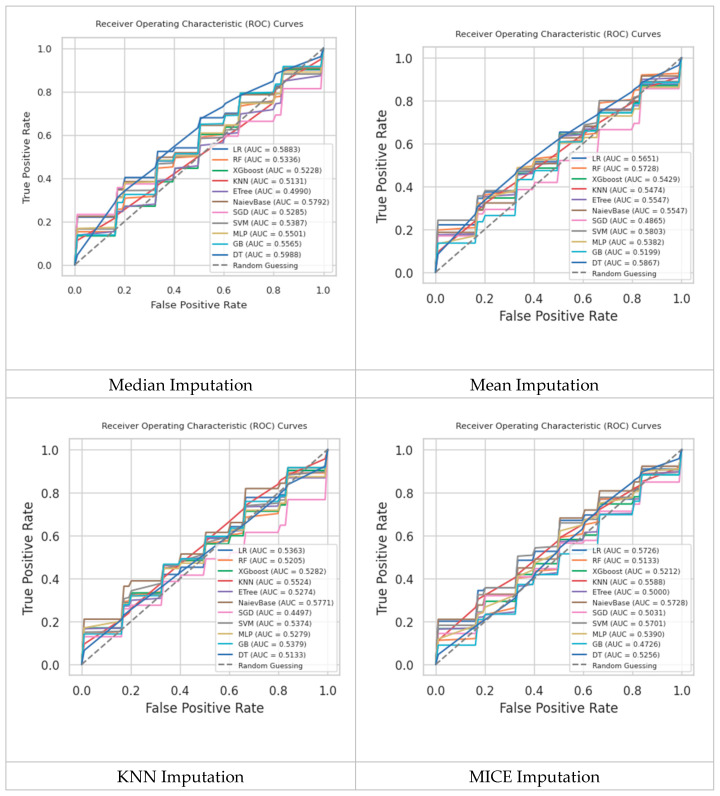
Comparison of the trained ML models’ results using RUS and different imputation techniques.

The ROC-AUC scores of all classification models using the four imputation techniques and the RUS are shown in Figure 7. Generally speaking, the figure demonstrates that the choice of imputation method and the application of ensemble classifiers significantly impact the performance of machine learning models in predicting kidney stones. Ensemble methods like random forest, XGBoost, and ETree consistently outperform others, highlighting their ability to handle complex datasets and improve classification accuracy. As can be seen, across all imputation methods, ensemble classifiers like random forest, XGBoost, and extremely randomized trees consistently achieve the highest ROC-AUC scores. This suggests that these models are better at capturing the complex relationships within the dataset. The imputation technique significantly affects the performance of the classifiers. Advanced imputation methods like MICE generally lead to better model performance compared to simpler methods like mean and median imputation. KNN imputation also shows competitive performance, indicating that considering the local structure of the data can be beneficial. The robustness of ensemble methods is evident from their consistent performance across different imputation techniques. These models are better at handling variability and missing data in the dataset. 

Moreover, the comparison underscores the critical role of the data preprocessing phase. Proper handling of missing data and class imbalance (through imputation and RUS) can significantly enhance the predictive power of models. 

**Figure 8:**
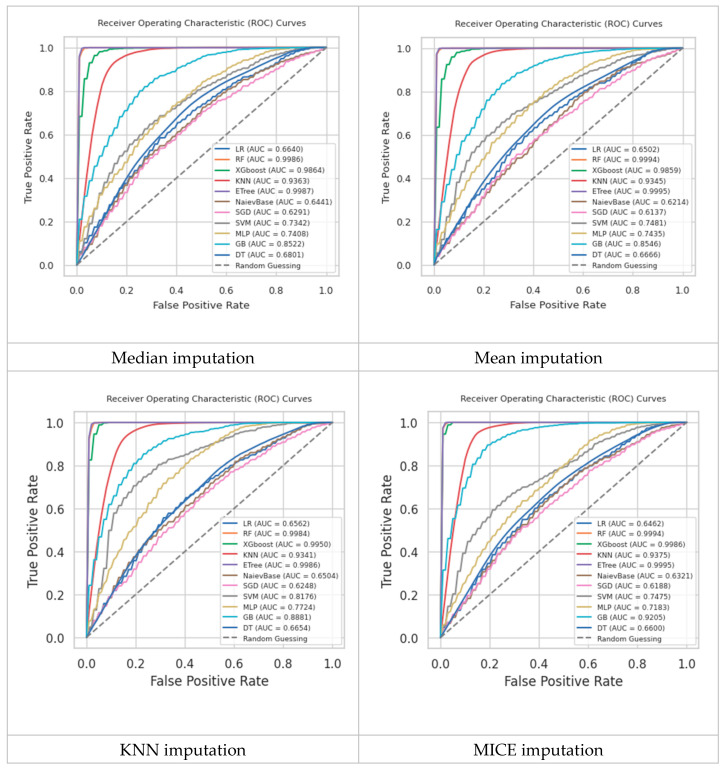
Comparison of the trained ML models’ results using ROS and different imputation techniques.

Figure 8 shows the ROC-AUC scores of all classification models using all imputations and ROS. Notably, KNN Imputation shows improved AUC scores for many classifiers compared to median and mean imputations, including KNN itself and GB (gradient boosting). This suggests that considering local data structure (as KNN imputation does) might help these models better capture the underlying patterns. The performance of other models, such as LR, SVM, and SGD, varies more significantly with changes in imputation technique. This variability can be attributed to how these models are affected by the underlying data distribution, which is altered by different imputation methods. RF and ETree again perform excellently, reinforcing their effectiveness across varied data treatments. Therefore, the choice of imputation method significantly affects classifier performance. Advanced techniques like MICE generally provide better results for most classifiers, suggesting that more sophisticated handling of missing data can lead to improvements in model accuracy. Thus, selecting the appropriate imputation technique can be crucial, especially for less robust models. KNN and MICE imputations show potential for better handling the complexities of real-world data, which might not always be well-represented by simpler imputation methods like the mean or median.

Table 2 shows the comparison results of all classification models regarding accuracy, precision, recall, and F1Score using MICE imputation and ROS. Different classifiers exhibit varying trade-offs between precision and recall. For instance, NB achieves high recall but lower precision, making it suitable for identifying positive cases, even at the expense of more false positives. In contrast, KNN achieves perfect precision but lower recall, indicating a conservative approach that minimizes false positives but may miss some true positives. The consistent performance of models like SVM and MLP across different imputation techniques highlights their stability and reliability. Although they do not outperform ensemble methods, their balanced metrics make them dependable choices. Moreover, their performance is less sensitive to the choice of imputation method, which can be advantageous when dealing with datasets of varying quality. As presented in the table, although a single DT could not model the underlying patterns in the training set, the ensemble of decision trees in RF, XGBoost, and ETree achieved high performance in all measures. This emphasizes the importance of applying ensemble learning to overcome all challenges associated with this dataset. Most importantly, as ensemble models fostered diversity, they were able to discover critical features and nonlinear relations between the features. 

In summary, the results underscore the critical importance of data preprocessing. Effective handling of missing data through robust imputation techniques and addressing class imbalance can significantly enhance model performance. Ensemble models, with their superior ability to manage the complexity of our dataset, and advanced imputation methods like MICE, which better retain data integrity, are key to achieving accurate and generalizable predictions. 

## 5. Conclusions

Despite advances in medical technology and treatment options, detecting and diagnosing renal stones remain challenging for healthcare professionals. In this paper, we explored the potential of ML methods in identifying the presence of renal stones based on laboratory results. Our study aimed to develop an accurate and non-invasive method for detecting renal stones, which would help healthcare professionals make an earlier diagnosis and prevent further complications. We collected and constructed a dataset of laboratory test results in collaboration with a local hospital in Jeddah, Saudi Arabia. To the best of our knowledge, this is the largest dataset used for this purpose. Developing a reliable and comprehensive database consisting of relevant laboratory results is critical to the success of ML methods.

Moreover, preprocessing medical data is vital to ensure the accuracy and reliability of ML models. This paper demonstrated that ML techniques could accurately detect the presence of renal stones based on laboratory test results, highlighting the importance of selecting the appropriate data balancing and imputation approaches. Among the difficulties faced in this work were cleaning and organizing the dataset, as the data were raw and required many steps to become suitable for processing and modeling. Additionally, dealing with an imbalanced dataset was challenging while training the model. We found that the number of patients with kidney stones in the study was much lower than those without. To resolve this issue, we evaluated different sampling techniques and examined several imputation approaches and classification models to identify the best-performing approach. There are several methods for dealing with missing data in the literature. In this work, we demonstrated that with proper preprocessing stages, including imputation, feature scaling, and sampling, ML models, primarily ensemble tree-based learners, can predict the presence of kidney stones with high accuracy using only lab test results.

We also demonstrated that training an ensemble of classifiers helps capture complex patterns, overcome data inconsistency, and improve the performance of all algorithms. For example, the DT classifier achieved 0.62 accuracy using MICE and ROS compared to 0.99 accuracy of RF. It is also worth noting that single classifiers performed slightly better than ensemble models using RUS. However, neither model achieved good results, likely because ensemble models overfitted the training set when the size of the training set was relatively small. Thus, a large dataset is needed. Moreover, the recall or sensitivity of RF and ETree ensemble models using ROS were above 0.95, demonstrating their ability to detect positive cases accurately. Oversampling the training set yielded significant learning improvement in all predictors. For example, the performance of the GB classifier using undersampling and KNN imputation was slightly better than random guessing, with an AUC of 0.53. In contrast, the GB classifier achieved an AUC of 0.88 using the same imputation technique but with oversampling. This highlights the need for a large dataset of lab test results to leverage the power of ML algorithms and help urologists and healthcare professionals make an earlier diagnosis, reduce costs, and prevent further complications.

Kidney stone disease has been mainly diagnosed using CT scans, but this study shows the potential of predicting kidney stones using only laboratory tests. This will help urologists and healthcare professionals make an earlier diagnosis and prevent further complications. The key contribution of this study lies in demonstrating that ML models, particularly ensemble tree-based learners, can achieve high accuracy and reliability using only lab test results. This approach offers a non-invasive, cost-effective alternative to traditional CT scans, potentially transforming how kidney stones are diagnosed and managed.

Building on the findings of this study, we aim to further refine our predictive model to enhance its diagnostic capabilities. For example, we aim to incorporate additional biochemical markers and imaging data into the model. Advanced algorithms capable of handling multi-dimensional data will be explored to improve specificity in stone-type prediction. Moreover, we plan to develop a predictive model that utilizes patient demographic data, stone characteristics, and previous treatment responses. Thus, the goal is to predict treatment outcomes, enabling clinicians to tailor interventions more effectively and efficiently. 

## Figures and Tables

**Table 1:** Properties of the original SRSP dataset’s features.

Feature	Description	Mean	Std	Missing	Notes
Age	Age of the patient (years)	43.75	16.77	0	The dataset includes a wide age range from 4 to 105 years, with a mean age of 43.75 years. No missing values in this feature.
Gender	Gender of the patient (0 for female, 1 for male)	0.51	0.50	0	Gender is almost evenly distributed, with a slight male predominance. This binary feature has no missing values.
BMI	Body mass index (kg/m^2^)	29.59	6.49	912	BMI has a substantial number of missing values (912). The mean BMI suggests a slightly overweight population.
Alkaline Phosphatase	Enzyme level in the blood (U/L)	95.00	67.48	1742	A high level of missing data (1742). The mean value indicates the average level of alkaline phosphatase in the blood, with a wide standard deviation reflecting variability.
Blood Urea Nitrogen Serum	Nitrogen level in the blood (mmol/L)	4.55	2.35	1271	This feature has many missing values (1271). The average value indicates normal kidney function, but there is considerable variability.
Calcium Serum	Calcium level in the blood (mmol/L)	2.24	0.23	2104	Very high missing data (2104). The mean and standard deviation suggest normal calcium levels with low variability.
Creatinine Serum	Creatinine level in the blood (µmol/L)	73.41	29.13	155	Few missing values (155). The mean value suggests normal renal function, but the high standard deviation indicates significant variability among patients.
Culture Urine	Urine culture result (0 for negative, 1 for positive)	0.29	0.46	2002	A high number of missing values (2002). Most samples are negative for urine culture, indicating infections are relatively rare.
Magnesium (Mg) Serum	Magnesium level in the blood (mmol/L)	0.80	0.11	2116	Almost complete missing data (2116). The mean value indicates typical magnesium levels with low variability.
Urine Nitrite	Nitrite presence in urine (0 for negative, 1 for positive)	0.03	0.18	1713	Many missing values (1713). The low mean suggests most urine samples are nitrite-negative, indicating a low prevalence of bacterial infections.
pH	pH level of the urine	6.03	0.88	1713	Significant missing data (1713). The mean pH value indicates slightly acidic urine, which is within the normal range, with moderate variability.
Phosphorous Serum	Phosphorous level in the blood (mmol/L)	1.13	0.20	2111	Very high missing data (2111). The mean and standard deviation suggest normal phosphorus levels with low variability.
Red Blood Cells in urine	Red blood cells count in urine (cells/HPF)	11.66	44.49	1801	High missing values (1801). The mean indicates a low level with high variability, suggesting occasional presence of hematuria in some patients.
White Blood Cells in urine	White blood cells count in urine (cells/HPF)	8.46	22.63	1802	High missing values (1802). Similar to RBCs, the mean indicates a low level with high variability, suggesting the occasional presence of infection or inflammation.
Stone	Presence of kidney stones (0 for no, 1 for yes)	0.11	0.31	0	This is the target variable with no missing values. The mean indicates that 10.8% of the patients have kidney stones, which shows the class imbalance in the dataset.

**Table 2:** Comparison of the trained ML models’ results using MICE and ROS.

Classifier	Accuracy	Precision	Recall	F1 Score
NB	0.48	0.49	0.90	0.63
LR	0.52	0.52	0.62	0.57
DT	0.62	0.67	0.50	0.57
KNN	0.80	1.00	0.60	0.75
SGD	0.49	0.49	0.62	0.55
SVM	0.56	0.56	0.56	0.56
MLP	0.66	0.69	0.56	0.62
RF	0.99	1.00	0.98	0.99
GB	0.79	0.89	0.67	0.76
XGBoost	0.90	0.96	0.93	0.87
ETree	0.92	1.00	0.96	0.92

## Data Availability

The data presented in this study are available on request from the corresponding author. The data are not publicly available due to restrictions, such as containing information that could compromise the privacy of research participants.
